# *Aspergillus fumigatus* Triggers Inflammatory Responses by Stage-Specific β-Glucan Display

**DOI:** 10.1371/journal.ppat.0010030

**Published:** 2005-11-18

**Authors:** Tobias M Hohl, Heather L. Van Epps, Amariliz Rivera, Laura A Morgan, Patrick L Chen, Marta Feldmesser, Eric G Pamer

**Affiliations:** 1 Infectious Diseases Service, Department of Medicine, Memorial Sloan-Kettering Cancer Center, New York, New York, United States of America; 2 Immunology Program, Sloan-Kettering Institute, Memorial Sloan-Kettering Cancer Center, New York, New York, United States of America; 3 Division of Infectious Diseases, Albert Einstein College of Medicine, Bronx, New York, United States of America; Johns Hopkins University, United States of America

## Abstract

Inhalation of fungal spores (conidia) occurs commonly and, in specific circumstances, can result in invasive disease. We investigated the murine inflammatory response to conidia of *Aspergillus fumigatus,* the most common invasive mold in immunocompromised hosts. In contrast to dormant spores, germinating conidia induce neutrophil recruitment to the airways and TNF-α/MIP-2 secretion by alveolar macrophages. Fungal β-glucans act as a trigger for the induction of these inflammatory responses through their time-dependent exposure on the surface of germinating conidia. Dectin-1, an innate immune receptor that recognizes fungal β-glucans, is recruited in vivo to alveolar macrophage phagosomes that have internalized conidia with exposed β-glucans. Antibody-mediated blockade of Dectin-1 partially inhibits TNF-α/MIP-2 induction by metabolically active conidia. TLR-2- and MyD88-mediated signals provide an additive contribution to macrophage activation by germinating conidia. Selective responsiveness to germinating conidia provides the innate immune system with a mechanism to restrict inflammatory responses to metabolically active, potentially invasive fungal spores.

## Synopsis

*Aspergillus fumigatus* is a mold that forms spores that are often inhaled by mammals. Humans with normal immune systems inhale several hundred *A. fumigatus* spores per day without developing detectable disease. Immunocompromised hosts, on the other hand, can develop invasive *A. fumigatus* infections. In these cases, inhaled spores germinate and form tissue-invasive hyphae that invade blood vessels and disseminate to remote tissues. The aim of this study was to investigate the normal inflammatory response to inhaled spores in mouse lungs. The researchers found that the earliest stage of spore germination, referred to as “swelling,” triggered the recruitment of inflammatory cells into the lung airways. Consistent with this finding, lung-derived cells stimulated with swollen spores secreted copious amounts of proteins that attract inflammatory cells. Analysis of spores revealed that swelling is accompanied by surface expression of β-glucan polymers. These carbohydrates, which are not present on the surface of mammalian cells, induce signaling by the mammalian Dectin-1 receptor and activate the expression of genes that promote the inflammatory response. The results suggest that mammalian lungs have evolved a mechanism to distinguish swollen and potentially threatening spores from innocuous, dormant spores.

## Introduction

The innate immune system confronts a wide spectrum of microbes, extending from the innocuous to the highly pathogenic [1–3]. Overly robust inflammatory responses can compromise host tissues and organ function, which, in the case of the lungs, can be severely debilitating or even lethal. In contrast, inadequate immune responses to pathogens can promote tissue invasion and systemic dissemination with equally dire consequences. Lung airways are perpetually exposed to inhaled particulate materials that include pollens, viruses, and bacterial and fungal spores. While many of these particles are innocuous, some spores have the potential to germinate and cause invasive diseases. Distinguishing these rare pathogenic microbes from the innocuous majority and calibrating inflammatory responses to the invasive potential of the microbe are fundamental challenges faced by the pulmonary innate immune system.

*Aspergillus fumigatus* is a mold that forms conidia with a diameter of 2–3 μm [4]. Owing to their small size, *A. fumigatus* conidia can bypass mucociliary clearance mechanisms and are inhaled into terminal airways and phagocytosed by alveolar macrophages (AMØs) [5,6]. Conidia are killed in a phagocyte oxidase-dependent manner [7]. Neutrophils recruited to the site of infection form a second line of defense against germinating conidia [5].

Conidial germination begins with swelling and progresses to germ tube formation and hyphal extension [8]. Genetic, acquired, or pharmacologically induced states that impair macrophage and neutrophil function enable in vivo conidial germination and the formation of fungal hyphae that can invade pulmonary tissues, enter the bloodstream, and disseminate to remote tissues [9].

Hyphae contain four major carbohydrate polymers: chitin, galactomannan, branched β–1,3/β–1,6 glucans, and linear β–1,3/β–1,4 glucans [10]. In contrast to hyphae, conidia have morphologically distinct features—an outermost proteinaceous rodlet layer [11] and an inner cell wall layer that is exposed during swelling [12], a process that can occur within macrophages [7]. The complex composition of the conidial cell wall is incompletely defined [13], and the identity of conidial surface molecules that induce proinflammatory cytokine/chemokine responses through cognate host cell receptors has not been determined.

Germline encoded pattern recognition receptors constitute a major surveillance and defense mechanism against microbial invaders [1–3,14]. The 11 mammalian toll-like receptors (TLRs) differ in their subcellular localization, tissue distribution, and ligand specificity, yet signal through a limited set of adaptor proteins that includes myeloid differentiation factor 88 (MyD88). TLR-mediated signals induce proinflammatory responses, activate antimicrobial effector functions, and stimulate adaptive immune responses. In the case of *A. fumigatus,* TLR-2, TLR-4, and MyD88 have been implicated in the recognition of conidia and hyphae [15–20]. TLR-2-, TLR-4-, and MyD88-deficient animals survive pulmonary *A. fumigatus* infection [21]; this finding implies the existence of MyD88-independent pathways in host defense against inhaled conidia.

Dectin-1 [22,23], a type II transmembrane protein that belongs to the NK-like C-type lectin-like receptor family, is a pattern recognition receptor for β–1,3/β–1,6-linked glucans [24]. Dectin-1 binds to the inert, *Saccharomyces cerevisiae*–derived, β-glucan-rich particle zymosan [24,25], *Candida albicans* [26]*,* murine sp. *Pneumocystis carinii* [27], and *Coccidioides posadasii* [28]. Dectin-1 activation results in phagocytic, proinflammatory, and antimicrobial responses [25,29–31]. The contribution of Dectin-1-mediated signals to innate immune defense against *A. fumigatus* infection remains undefined.

To characterize the innate inflammatory response following spore inhalation, we investigated cell recruitment into the airways of immune competent mice exposed to live and killed *A. fumigatus* conidia. The early pulmonary innate immune response to these two challenges is distinct; live conidia induce rapid neutrophil recruitment, while killed conidia are far less stimulatory. Live conidia display β-glucans on their cell surface during swelling and hyphal formation; this process triggers Dectin-1 recruitment to phagosomal membranes of AMØs in vivo. Exposure of macrophages to germinating conidia reveals a direct correlation between surface β-glucan display and inflammatory cytokine/chemokine induction*.* This induction is partially inhibited by antibody-mediated blockade of Dectin-1 signaling. The innate immune system, therefore, restricts responsiveness to conidia that are germinating and ignores the less threatening dormant conidia. This mechanism may limit pulmonary damage following microbial spore inhalation.

## Results

### Bronchoalveolar Cellular Infiltrates Vary in Response to Live and Heat-Killed Conidia

To examine in vivo inflammatory responses to metabolically active and inert *A. fumigatus* conidia, 10^7^ live or heat-killed conidia were administered intratracheally to immune competent mice. Cell recruitment into the bronchoalveolar lavage (BAL) fluid collected 24 h later was quantified and analyzed by flow cytometry. In a representative experiment, BAL fluid recovered from mice that were infected with live conidia contained 3.0 ± 1.2 × 10^6^ cells (Figure 1A) with a predominant neutrophilic infiltrate consisting of Ly6G^hi^, CD11b^+^, and CD11c^−^ cells (Figure 1B; unpublished data). In comparison, heat-killed conidia induced modest cell recruitment into the BAL (0.36 ± 0.1 × 10^6^ cells), with a predominance of CD11c^+^, Ly6G^int^ AMØs. BAL fluid collected from mice administered PBS-Tween (vehicle) contained only 0.97 ± 0.17 × 10^5^ cells with >90% AMØs. The 8- to 9-fold increase in total BAL cells in mice administered live rather than heat-killed conidia was almost entirely attributable to the increased influx of neutrophils.

### Macrophages and Dendritic Cells Secrete TNF-α and MIP-2 in Response to Live but Not Heat-Killed Conidia

Neutrophil recruitment to sites of infection and inflammation depends, in part, on chemokine signals. In immune competent mice, the chemokine receptor CXCR2 is critical for host survival following *A. fumigatus* pulmonary challenge [32]. CXCR2 binds the neutrophil chemoattractants macrophage inflammatory protein-2 (MIP-2) and KC. The proinflammatory cytokine tumor necrosis factor-α (TNF-α) is produced in response to *A. fumigatus* challenge in vivo and influences multiple facets of the early innate immune response to inhaled conidia [33,34].

To determine whether live conidia differ from heat-killed conidia in their ability to stimulate inflammatory cytokine and chemokine production, bone marrow-derived macrophages (BMMØs) were stimulated for 18 h with both types of conidia. Cell culture supernatants from BMMØs stimulated with live conidia contained >50-fold higher TNF-α and >10-fold higher MIP-2 concentrations than cells exposed to heat-killed conidia (Figure 1C). Paraformaldehyde inactivation of resting conidia yielded similar results (unpublished data).

Since dendritic cells (DCs) [35,36] and AMØs [5] are implicated in innate immune defense against *A. fumigatus,* their responses to live and heat-killed conidia were investigated. While bone marrow-derived dendritic cells (BMDCs) (Figure 1D) stimulated with live conidia produced robust levels of TNF-α/MIP-2, AMØs were the most responsive cell type (Figure 1E). As noted for BMMØs, the BMDC and AMØ response to heat-killed conidia was markedly attenuated.

These experiments were performed in the presence of 0.5 μg/ml voriconazole to prevent fungal overgrowth of cell cultures. This drug concentration was chosen since it did not prevent conidial swelling or compromise viability during an overnight incubation ([37]; unpublished data). Hyphae were not observed under these conditions but formed rapidly if voriconazole was removed from the medium. The results from the macrophage/DC stimulation experiments thus indicate that conidial swelling is sufficient to induce inflammatory cytokine/chemokine production.

**Figure 1:**
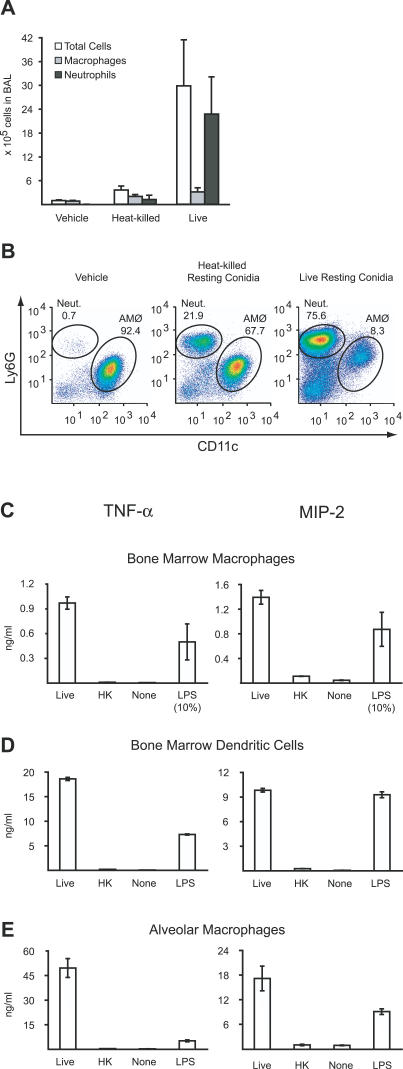
Live but Not Heat-Killed *A. fumigatus* Conidia Induce Inflammatory Responses (A and B) In vivo cellular recruitment into lung airways. (A) Absolute number of BAL cells, total neutrophils (Ly6G^hi^, CD11c^−^) in BAL, and total macrophages (CD11c^+^, Ly6G^int^) in BAL for C57BL/6 mice 24 h after intratracheal instillation of PBS-Tw (vehicle), 10^7^ heat-killed, or 10^7^ live conidia. The bar graphs show the average cell numbers + standard deviation from four mice per group. One of three representative experiments is shown. (B) Flow cytometric analysis of live BAL cells stained for Ly6G and CD11c. Gates representing neutrophils (Ly6G^hi^, CD11c^−^) and AMØs (CD11c^+^, Ly6G^int^) are shown and the frequencies of these cell populations are indicated in representative BAL samples. (C–E) Ex vivo TNF-α/MIP-2 secretion by BMMØs (C), BMDCs (D), and AMØs (E) stimulated with live or heat-killed *A. fumigatus* conidia, PBS-Tw, or LPS (100 ng/ml) for 18 h in medium containing 0.5 μg/ml voriconazole. TNF-α/MIP-2 concentrations in the culture supernatants were determined by ELISA. In (C), the values for TNF-α/MIP-2 secretion induced by LPS were reduced by a factor of ten. The bar graphs represent the average cytokine production ± standard deviation by cells in 3–4 wells per condition.

### Heat-Killed Conidia That Have Initiated the Germination Process Recruit Neutrophils into the Airways and Induce TNF-α/MIP-2 Secretion

Heat inactivation arrests conidial swelling and germination at a defined time point. To examine the effect of conidial swelling on in vivo inflammatory responses, conidia were swollen, heat-killed to prevent further germination, and administered intratracheally into mice. In contrast to conidia killed in the resting state (see Figure 1B), conidia that were swollen prior to heat inactivation induced an inflammatory cell influx comparable both in number and cell type to that induced by an inoculum of live conidia (Figure 2A and 2B). This result indicates that the pulmonary innate immune system selectively recognizes conidia that have initiated germination; once swollen, conidial viability is no longer critical for the initiation of inflammatory responses.

Conidia heat-killed prior to significant conidial swelling (3 h incubation in RPMI medium) did not induce TNF-α/MIP-2 secretion by BMMØs (Figure 2C). In contrast, fungal preparations containing heat-killed swollen conidia (5 h incubation in RPMI medium) induced approximately the same amount of TNF-α/MIP-2 as live, resting conidia (see Figure 1C). As germination proceeds for longer time periods prior to heat-killing (7 h incubation in RPMI medium), TNF-α/MIP-2 production increases dramatically. These results indicate that conidial germination alters the fungal cell surface and renders it far more inflammatory.

### β-Glucans Become Surface-Exposed during Swelling and Germ Tube Formation

β-glucans have been implicated as fungus-derived targets of mammalian innate immune receptors [29]. We generated a β-glucan specific monoclonal antibody (mAb 744) by immunizing mice with *A. fumigatus* conidia. This antibody binds immobilized laminarin (M_w_ < 10,000), a β–1,3 glucan polymer with β–1,6 interstrand linkages (Figure 3A). The mAb 744 binding is disrupted by addition of laminarinase, defining β-glucan as the cognate antigen. Confocal microscopy of unfixed fungal cells labeled with mAb 744 indicated that β-glucan cell surface immunoreactivity was prominent in swollen conidia and in early germlings, particularly at sites of initial hyphal extension (Figure 3B).

Resting conidia failed to stain with mAb 744, demonstrating that β-glucans are exposed only on germinating spores. No fungal cell surface fluorescence was observed if mAb 744 was depleted by a prior incubation with the insoluble β-glucan-rich particle zymosan (Figure 3C) or replaced with an isotype control antibody (Figure 3D). These results demonstrate that β-glucans are exposed on the conidial surface in a stage-specific fashion, either through de novo synthesis or exposure of a concealed layer.

**Figure 2:**
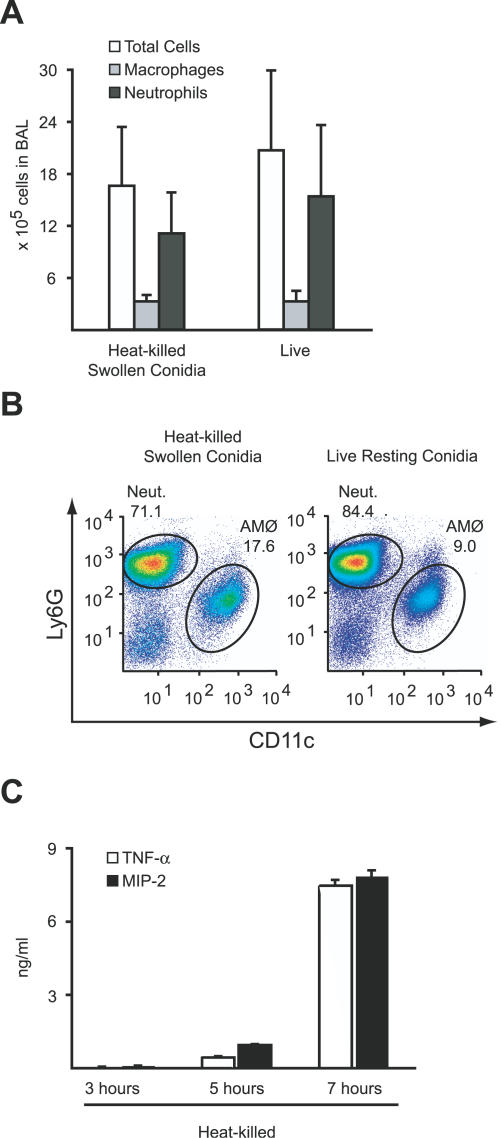
Germinating *A. fumigatus* Conidia Are Highly Inflammatory (A) Absolute number of BAL cells, total neutrophils in BAL, and total macrophages in BAL for C57BL/6 mice 24 h after intratracheal instillation with either heat-killed swollen conidia or live conidia. The bar graphs show the average cell numbers + standard deviation from four mice per group. (B) Flow cytometric analysis of live BAL cells stained for Ly6G and CD11c as in Figure 1B. (C) *A. fumigatus* conidia were allowed to initiate germination for 3, 5, or 7 h, heat-killed, and added to BMMØ cultures for 18 h. TNF-α/MIP-2 concentrations in the supernatants were determined by ELISA. The bar graphs represent the average ± standard deviation of three wells per condition. One of four experiments is shown.

### β-Glucan Exposure Alters Dectin-1 Intracellular Distribution in AMØs

To determine the effect of β-glucan exposure on the in vivo distribution of Dectin-1 in AMØs, mice were inoculated intratracheally with either heat-killed swollen conidia (Figure 4A–4D) or heat-killed resting conidia (Figure 4E–4H). AMØs were harvested 45 min later by BAL and processed immediately for immunofluorescence microscopy. Immunostaining for Dectin-1 revealed that phagocytosis of heat-killed swollen conidia (β-glucan surface-positive) (Figure 4B) triggered recruitment of Dectin-1 to the phagosomal membrane, resulting in a ring pattern of fluorescence surrounding the ingested conidia (Figure 4C). Merging the fluorescence images confirmed that the red Dectin-1 fluorescence signal surrounded (and partially overlapped) the green fluorescence signal from the labeled, surface-exposed conidial β-glucans (Figure 4D). Voriconazole treatment and heat-killing did not appreciably influence β-glucan immunostaining on the surface of swollen conidia (see Figures 3B and 4B; unpublished data).

Heat-killed resting conidia are β-glucan surface-negative (Figure 4F), in contrast to heat-killed *C. albicans* yeast cells [26]. Phagocytosis of heat-killed resting conidia did not result in Dectin-1 redistribution around the ingested conidia (Figure 4G). These results suggest that Dectin-1 associates with inhaled *A. fumigatus* conidia in a β-glucan-dependent manner in vivo.

### Conidia Activate Two Parallel Macrophage Signaling Pathways That Lead to TNF-α and MIP-2 Production

To examine whether Dectin-1 mediates proinflammatory responses to *A. fumigatus,* TNF-α/MIP-2 secretion by BMMØs in response to live conidia was measured in the presence of the anti-Dectin-1 blocking antibody 2A11. Cytochalasin D was added to these assays to prevent antibody degradation or dissociation in the vacuolar compartment. In comparison to cells incubated with an IgG_2b_ control antibody, 2A11 administration reduced TNF-α/MIP-2 secretion in BMMØs by approximately 40%–50% (Figure 5A). On the basis of this result, Dectin-1-mediated signaling accounts for nearly one-half of the TNF-α/MIP-2 released by BMMØs.

To assess the role of MyD88-dependent TLR-signaling in TNF-α/MIP-2 release, MyD88^−/−^ BMMØs were stimulated with live conidia. In MyD88^−/−^ BMMØs, TNF-α/MIP-2 secretion was consistently reduced by approximately 40%–50% as compared to wild-type (WT) (MyD88^+/+^) control cells (Figure 5B). Since TLR-2 has been implicated in conidial recognition [17–19] and may interact with Dectin-1 [25,29] TNF-α/MIP-2 release was also examined in TLR-2^−/−^ BMMØs. TLR-2^−/−^ BMMØs released approximately 20% less TNF-α/MIP-2 than WT (TLR-2^+/+^) BMMØs (Figure 5B). This result suggests that TLR-2 engagement does not fully account for the MyD88-dependent TNF-α/MIP-2 release triggered by *A. fumigatus* conidia.

Since Dectin-1- and MyD88-dependent signals each account for 40%–50% of TNF-α/MIP-2 release by BMMØs, MyD88^−/−^ cells were treated with the anti-Dectin antibody and stimulated with conidia to determine whether Dectin-1-mediated TNF-α/MIP-2 release relies on the presence of MyD88. MyD88^−/−^ BMMØs incubated with anti-Dectin-1 antibody secreted approximately 70% less TNF-α/MIP-2 than MyD88^−/−^ BMMØs treated with an isotype control antibody (Figure 5C). The extent of TNF-α/MIP-2 inhibition by Dectin-1 blockade was similar in MyD88^−/−^ and WT BMMØs. This result suggests that conidia activate independent pathways that signal through MyD88 and Dectin-1 and that these pathways are additive with respect to TNF-α/MIP-2 secretion. A similar independence and summed contribution of MyD88- and Dectin-1-mediated signals to TNF-α/MIP-2 release was observed if either voriconazole (to exclude a drug-mediated effect) or cytochalasin D (to exclude an effect of blocking phagocytosis) was omitted from the samples (unpublished data).

**Figure 3:**
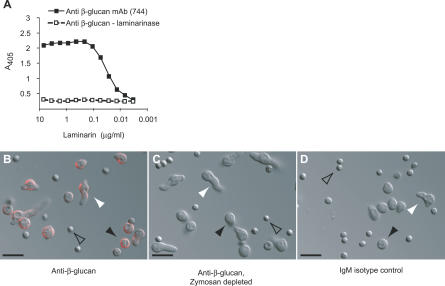
Germinating but Not Resting *A. fumigatus* Conidia Display β-Glucans on the Cell Surface (A) The mAb 744 detects β-glucans. Decreasing laminarin concentrations were coated onto ELISA plates in the presence or absence of laminarinase. 10 μg/ml mAb 744 was added to the wells and detected using an alkaline phosphatase conjugated anti-mouse IgM. (B–D) *A. fumigatus* conidia were incubated in RPMI for 7 h, stained with mAb 744 (B), mAb 744 depleted by zymosan (C), or IgM control antibody (D), followed by Alexa Fluor 594-anti-mouse IgM, and examined by confocal microscopy. Representative DIC/epifluorescence confocal images of resting conidia (open arrowheads), swollen conidia (black arrowheads), and early germlings (white arrowheads) are shown in overlay. Scale bar = 10 μm.

The additive effect of Dectin-1- and MyD88-dependent signaling accounted for approximately 80%–85% of TNF-α/MIP-2 secretion by BMMØs (Figure 5C). In AMØs, the combination of Dectin-1 blockade and MyD88 deficiency led to a similar reduction in TNF-α/MIP-2 secretion, approximately 70%–80% as compared to the control condition with WT cells incubated with an isotype control antibody (Figure 5D).

**Figure 4:**
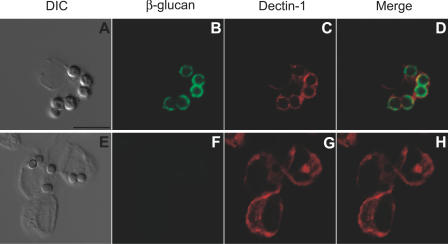
Dectin-1 Is Recruited to AMØ Phagosomes Containing Swollen Conidia with Surface-Exposed β-Glucans (A–H) AMØs were harvested from C57BL/6 mice 45 min after intratracheal instillation of 10^7^ heat-killed swollen conidia (A–D) or heat-killed resting conidia (E–H), processed at 4 °C, and immunostained for ingested conidial β-glucans with mAb 744, followed by a FITC-coupled anti-mouse IgM (B and F) and for Dectin-1 (C and G) with goat anti-Dectin polyclonal antibodies, followed by Alexa Fluor 594-coupled anti-goat IgG. AMØs were examined by DIC microscopy (A and E) and epifluorescence confocal microscopy (B–D and F–H). (D and H) Merged images of β-glucan and Dectin-1 immunofluorescence. Scale bar = 10 μm.

## Discussion

In this study, we show that innate immune responses to *A. fumigatus* conidia depend on host recognition of morphologic changes that occur during the first step of germination, conidial swelling. Our data indicate that remodeling and expansion of the cell wall during conidial swelling results in the exposure of β-glucan polymers on the fungal cell surface. Swollen conidia display surface β-glucan and associate with Dectin-1 in AMØs isolated from in vivo challenged mice. Two parallel innate immune signaling pathways respond to this process. One pathway signals through the TLR adaptor protein MyD88, the other through the β-glucan receptor Dectin-1.

**Figure 5:**
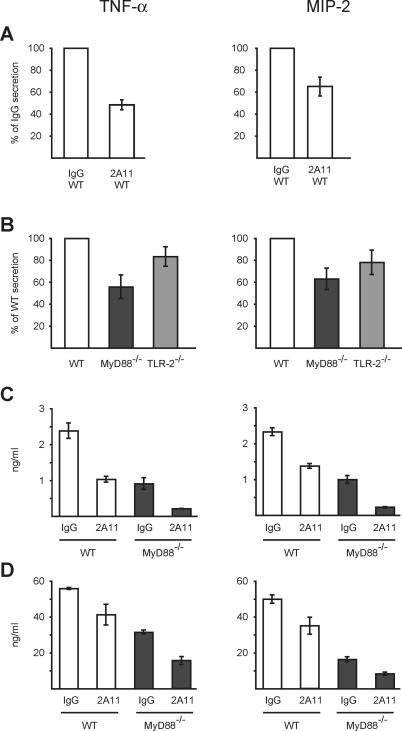
Dectin-1- and MyD88-Mediated Signals Are Induced by *A. fumigatus* Conidia (A–D) WT (white bars, [A–C]), MyD88^−/−^ (dark grey bars, [B–D]), and TLR-2^−/−^ (light grey bars, [B]) BMMØs (A–C) or AMØs (D) were stimulated with conidia for 18 h in medium containing 0.5 μg/ml voriconazole, and TNF-α/MIP-2 secretion was measured by ELISA. (A,C,D) BMMØs (A and C) and AMØs (D) were incubated with 2A11 (anti-Dectin-1 mAb) or an isotype control antibody in the presence of 2 μM cytochalasin D. (A and B) ELISA results from test conditions were averaged among three to five experiments and expressed as a percentage ± standard deviation of the averaged value obtained for the control condition. (C and D) show representative experiments. All experiments were performed with three to six replicates per condition.

Several features of this response are striking. First, neither innate immune response pathway is activated by dormant conidia; each responds only to conidia that have initiated the germination process. We propose that restricted recognition of germinating conidia provides a mechanism to focus pulmonary inflammatory responses on spores that are most likely to cause invasive disease. Second, the contributions of the Dectin-1- and MyD88-mediated signaling pathways are, in the case of *A. fumigatus* conidia, additive. The ability of AMØs to generate Dectin-1-dependent, MyD88-independent inflammatory responses to germinating conidia may, in part, provide an explanation for the survival of TLR-2- and MyD88-deficient mice in a pulmonary infection model [21].

The relationship between MyD88- and Dectin-1-dependent signaling has not been examined previously in the host response to intact fungi. Studies with the β-glucan-rich particle zymosan revealed that TNF-α secretion by macrophages is fully dependent on the presence of TLR-2 and MyD88 and is blocked by the addition of glucan phosphate, a soluble high-molecular-weight β-glucan [25,29]. Experiments with transfected cell lines found that co-expression of Dectin-1- and TLR-2- enhanced zymosan-dependent transcriptional responses [25]. Zymosan also binds the extracellular domain of TLR-2 in vitro [38]. Taken together, these results suggest that Dectin-1 and TLR-2/MyD88 collaborate to mount zymosan-dependent inflammatory responses. In our experimental system, Dectin-1- and MyD88-mediated TNF-α/MIP-2 secretion did not depend on the functional presence of the other signaling molecule.

Our results, to our knowledge, differ from previous work examining TLR- and MyD88-dependent signaling in response to conidia and hyphae. This may be, in part, due to differences in TLR/MyD88- and Dectin-1-dependent signaling by macrophages isolated from different anatomical sites. Mambula et al. found that in peritoneal macrophages MyD88-dependent signals account for approximately 90% of TNF-α secreted in response to live resting and heat-killed swollen conidia [17]. In contrast, our results suggest that MyD88-dependent signals account for roughly 50% of the TNF-α secreted by BMMØs and AMØs in response to live, resting conidia. Macrophages from different sources may also react uniquely to killed, resting conidia. For example, Marr et al. also observed that BMMØs do not produce TNF-α in response to heat-killed resting conidia [20], while two other studies demonstrated that peritoneal macrophages secrete TNF-α in response to heat- or ethanol-killed conidia in a largely TLR-4-dependent fashion [18,19]. We have investigated AMØs given their central role as first responders to inhaled conidia. Unlike peritoneal macrophages, AMØs and BMMØs kill conidia and prevent germination [7,20,39].

Conidial ligands that activate TLR/MyD88-dependent signals remain unknown and may involve molecules derived from carbohydrates, proteins, or lipids. The complex interaction between conidia and mammalian cells involves multiple sets of ligand-receptor interactions beyond the β-glucan/Dectin-1 and the unknown ligand(s)-TLR/MyD88 pairs. Experiments using soluble carbohydrates or antibodies as blocking agents have implicated a number of receptors in conidial binding or internalization [35,40,41]; these include a mannosyl-fucosyl receptor (in murine AMØs), a β-glucan inhibitable receptor (in human monocytes), the macrophage mannose receptor, the DEC205 lectin, and the CD11b/CD18 integrin (all in murine pulmonary DCs). Conidial galactomannan binds the soluble pattern recognition receptor pentraxin 3 [42], a C-type lectin of galactomannan-specificity on Langerhans cells [43], as well as DC-specific intercellular adhesion molecule 3-grabbing nonintegrin (DC-SIGN) on human DCs [36]. However, it remains unknown whether DC-SIGN or any of these other cell surface receptors implicated in conidial binding or internalization contribute to inflammatory cytokine/chemokine induction and neutrophil recruitment.

Human fungal pathogens propagate and grow using different mechanisms. For example, the yeast *C. albicans* divides by separation of mother and daughter cells and can switch between yeast and filamentous growth. It has been recently demonstrated that the process of *C. albicans* cell division creates bud and birth scars with exposed β-glucans [26]. Unlike filamentous growth, *C. albicans* yeast growth is thus susceptible to Dectin-1-dependent recognition and antifungal responses [26].

*Paracoccidioides brasiliensis,* a thermal dimorph, forms conidia that are inhaled and transform into pathogenic yeast forms within the host. The dimorphic transition is associated with a change in the polymer linkage from β-glucan to α-glucan in the cell wall [44]. In contrast, the mold *A. fumigatus* forms conidia that swell and germinate prior to hyphal extension; our study indicates that this process exposes β-glucan polymers on the surface of swollen conidia. The appearance of surface-exposed β-glucans at specific stages of fungal growth and division as well as their conserved presence among fungal organisms renders these carbohydrate polymers ideal recognition molecules for innate immune receptors that trigger antifungal responses.

*C. albicans, P. jiroveci, C. posadasii,* and *A. fumigatus* vary significantly in terms of human disease and host susceptibility, tissue tropism and damage, and metastatic potential [15]. However, innate immune defenses against these organisms are mobilized, at least in part, through the recognition of exposed β-glucans by Dectin-1-dependent pathways. Despite their common β-glucans, yeasts, molds, and zymosan differ in terms of other surface and cell-wall components. It is likely that these disparities account for distinct innate immune responses to different fungal organisms and components. The identification of both common and distinct fungal components that activate innate immune responses will be an intense focus of further research. In turn, the repertoire of signaling pathways activated in leukocytes and other host cells will undoubtedly yield experimental strategies to modulate inflammatory states and responses.

## Materials and Methods

### Chemical reagents and antibodies.

Chemical reagents were purchased from Sigma-Aldrich (St. Louis, Missouri, United States) unless noted otherwise. All cell culture reagents were purchased from Invitrogen (Carlsbad, California, United States).

The mAb 744 (IgMκ) was generated by the fusion of splenocytes from a BALB/c mouse immunized with heat-killed resting conidia of *A. fumigatus* strain 293 to the Ag8–653 myeloma cell line. Hybridomas were screened for binding to the conidial surface by ELISA and were cloned twice on soft agar.

The anti-Dectin-1 mAb 2A11 (rat IgG_2b_) was obtained from G. Brown [24] for use in pilot experiments. The 2A11 mAb is commercially available from Cell Sciences (Canton, Massachusetts, United States). Affinity-purified goat anti-Dectin and goat IgG control antibodies were from R&D Systems (Minneapolis, Minnesota, United States), rat IgG_2b_ isotype controls and antibodies for flow cytometry (see below) were from BD Biosciences Pharmingen (San Diego, California, United States), FITC-coupled donkey anti-mouse IgM were from Jackson Immunoresearch (West Grove, Pennsylvania, United States), and Alexa Fluor 594-coupled goat anti-mouse IgM and Alexa-Fluor 594-coupled donkey anti-goat IgG were from Molecular Probes (Eugene, Oregon, United States).

### Aspergillus growth and culture.

*A. fumigatus* strain 293 was grown on Sabouraud dextrose agar slants incubated at 37 °C for 5–8 d. Conidia were dislodged from slants by gentle tapping and then resuspended in PBS containing 0.025% (w/v) Tween-20 (PBS-Tw), filtered twice through a 40-μm nylon cell strainer (BD Falcon), and, as required, heat-killed at 100 °C for 30 min in a heating block or at 121 °C for 15 min in an autoclave.

Conidia were incubated at 37 °C at a concentration of 5 × 10^6^ conidia/ml in RPMI for the indicated times. Preparations incubated for 5 h contained swollen conidia. Preparations incubated for 7 h included early germlings with <10 μm hyphal extensions. For in vivo experiments shown in Figures 2A and 4A–D, homogeneous preparations of swollen conidia were prepared by incubating conidia in RPMI containing 0.5 μg/ml voriconazole (Pfizer, New York, New York, United States) for 12 h at 37 °C. Fungal cells were washed twice, adjusted to 2 × 10^8^/ml in PBS-Tw, and stored at 4 °C for use within 48 h. The conidial concentration and the efficiency of heat-killing was verified by plating serial dilutions on Sabouraud dextrose agar.

### Animal care.

C57BL/6 mice were purchased from Jackson Laboratory (Bar Harbor, Maine, United States). MyD88^−/−^ and TLR-2^−/−^ mice were backcrossed at least ten generations on the C57BL/6 background and maintained under pathogen-free conditions in the animal care facilities at Memorial Sloan-Kettering Cancer Center (New York, New York, United Sates). In vivo studies were performed in accordance with institutional guidelines regarding animal care.

### Intratracheal injections and flow cytometry.

A blunt-end, 20-gauge needle was used to administer 10^7^ conidia (in 50 μl PBS-Tw) intratracheally to mice anesthetized with isoflurane and immobilized in an upright position. Mice were sacrificed either 45 min or 24 h later for recovery of BAL cells though eight stepwise 0.5-ml rinses using a sterile Angiocath plastic catheter (Becton-Dickinson [BD], Palo Alto, California, United States) inserted into the trachea. The BAL specimens were harvested with PBS, 5% FCS for flow cytometry, or RP10 (see below) for immunofluorescence experiments.

BAL cells were counted using a hemocytometer, incubated in 5 μg/ml anti-F_c_γ III/II receptor (clone 2.4G2), stained with anti-Ly6C-FITC (AL-21), anti-Ly6G-PE (1A8), anti-CD11b-PERCP (M1/70), and anti-CD11c-APC (HL3) in FACS buffer (PBS, 1% FCS, and 0.05% NaN_3_), and analyzed on a BD LSR flow cytometer. AMØs stained with anti-Ly6G-PE gave rise to a Ly6G^int^ fluorescence signal that reflects autofluorescence of this cell type.

### Cell culture.

BMDCs were eluted from the tibias and femurs of 8- to 12-wk-old mice in RP10 (RPMI, 10% FCS, and 5 mM HEPES, 1.1 mM L-glutamine, 0.5 U/ml penicillin, 0.5 μg/ml gentamicin, 50 μg/ml streptomycin, and 50 μM 2-ME), filtered through a 100-μm nylon mesh, and cultured in RP20 (20% FCS) supplemented with 30% (v/v) L929 cell supernatant (source of M-CSF) at a density of 0.25–0.5 × 10^6^ cells/ml (in 100-mm cell culture dishes) for the generation of BMMØs or in RP10 and 2% (v/v) p815 cell supernatant (source of GM-CSF) at a density of 10^6^ cells/ml (in 24-well dishes) for the generation of BMDCs.

For BMMØ cultures, the medium was exchanged on day 3. Cells were plated in 0.2 ml RP10 at a density of 4–6 × 10^5^ cells/ml in 96-well plates on day 5–6 for conidial stimulation 6–24 h later. For BMDC cultures, non-adherent cells were gently removed on day 2 and fresh medium was added every 2 d. On day 5, the medium was exchanged to RP10 (1 ml) prior to conidial stimulation.

To obtain AMØs, the BAL fluid from 10–12 uninfected mice was pooled and centrifuged at 300 × *g* for 5 min. BAL cells were plated in 0.2 ml RP10 at a concentration of 3.5–5.0 × 10^5^ cells/ml in 96-well plates. Non-adherent cells were removed after overnight incubation, and AMØs were stimulated with conidia on the day following the harvest. AMØs were >95% pure as judged by microscopic analysis of cells spun on slides and stained with Diff Quik (Fisher Scientific, Pittsburgh, Pennsylvania, United States).

### Conidial stimulation of macrophages and DCs.

Conidia were added at a 10:1 cell ratio to BMMØs, BMDCs, and AMØs and incubated for 18 h in RP10 in the presence of 0.5 μg/ml voriconazole (Pfizer), as indicated, and supernatants were collected for ELISA. For antibody inhibition experiments, cells were incubated in 0.2 ml of RP10 containing 5–10 μg/ml 2A11 or isotype control antibody. Conidia were added 30 min later in a volume of 5 μl of PBS-Tw, and no further antibody was added to the samples. Pilot experiments showed maximal inhibition of conidial-dependent TNF-α/MIP-2 secretion if the 2A11 concentration was ≥ 2 μg/ml. Cytochalasin D was added to a final concentration of 2 μM 30 min prior to conidial stimulation, as indicated.

### ELISA.

Commercially available ELISA kits for TNF-α (BD Biosciences Pharmingen, San Diego, California, United States) and MIP-2 (R&D Systems) were used according to the manufacturers' instructions. The limit of detection was 15 pg/ml for both TNF-α and MIP-2.

To determine laminarin binding by mAb 744, a 10 μg/ml solution of laminarin from *Laminaria digitata* was added to wells of a 96-well EIA plate (Corning, Corning, New York, United States) and serially diluted 1:2 in buffer (PBS with 10 mM NaN_3_) across the plate. For laminarinase treatment, a 10 μg/ml solution of laminarin with 2 U/ml of laminarinase from *Penicillium spp*. was added to the wells as described above*.* After incubation at 37 °C for 20 h, the wells were washed, blocked for 1 h at 37 °C in PBS, 2% BSA, and 10 mM NaN_3_, and washed again. The mAb 744 was added to the wells at a final concentration of 10 μg/ml, and the plates were incubated for 1 h at 37 °C. After washing, a 1:1,000 dilution of alkaline phosphatase-conjugated anti-mouse IgM was added to wells and the ELISA developed with *p*-nitrophenyl phosphate as a substrate. The A_405_ was measured using a VersaMax Microplate Reader (Molecular Devices, Sunnyvale, California, United States).

### Immunofluorescence and confocal microscopy.

Fungal cells grown in 4-well glass chamber slides (Lab-Tek, Campbell, California, United States) were blocked in RP10 containing 0.025% Tween-20 and 0.05% NaN_3_ and stained with 10 μg/ml mAb 744 (or a mouse IgM isotype control) followed by 10 μg/ml Alexa Fluor 594-goat anti-mouse IgM. To deplete mAb 744 with zymosan, 1 mg of zymosan was added to 10 μg of mAb 744 in 1 ml of PBS and incubated at 4 °C for 30 min with gentle agitation. Zymosan was removed by centrifugation, and the resulting supernatant was used for immunofluorescence staining.

AMØs harvested by BAL were processed at 4 °C, centrifuged on 8-well glass chamber slides (10^5^ cells/well), fixed and permeabilized with ice-cold ethanol, blocked in PBS, 10% donkey serum, and 5 μg/ml F_c_ block, and decorated with goat anti-Dectin-1 and mAb 744. The samples were washed and incubated with Alexa Fluor 594-donkey anti-goat IgG and FITC-donkey anti-mouse IgM. Duplicate samples decorated with isotype control primary antibodies did not yield fluorescence signals. All samples were imaged in an upright Leica (Wetzlar, Germany) TCS SP2 AOBS confocal microscopy system using 488 and 594 nm laser lines with a 63x Leica HPX PL APO water-immersion objective (NA = 1,2).

## Abbreviations

AMØ: alveolar macrophage
BAL: bronchoalveolar lavage
BMDC: bone marrow-derived dendritic cell
BMMØ: bone marrow-derived macrophage
DC: dendritic cell
mAb: monoclonal antibody
MIP-2: macrophage inflammatory protein-2
MyD88: myeloid differential factor 88
TLR: toll-like receptor
TNF-α: tumor necrosis factor-α
WT: wild-type
